# Baicalin ameliorates the gut barrier function and intestinal microbiota of broiler chickens: Baicalin protects broiler’s gut barrier

**DOI:** 10.3724/abbs.2024029

**Published:** 2024-03-20

**Authors:** Shuangxiu Wan, Linzheng Wang, Zhili Hao, Lin Zhu, Xiaoxia Mao, Hongquan Li, Panpan Sun, Wei Yin, Kuohai Fan, Hailong Zhang, Beibei Li, Wansen Nie, Zongjie Li, Na Sun

**Affiliations:** 1 Shanxi Key Lab for Modernization of TCVM College of Veterinary Medicine Shanxi Agricultural University Taigu 030801 China; 2 College of Pharmacy Heze University Heze 274000 China; 3 College of Traditional Chinese Medicine Shandong University of Traditional Chinese Medicine Jinan 250035 China; 4 College of Veterinary Medicine Jilin University Changchun 130012 China; 5 Shanghai Veterinary Research Institute Chinese Academy of Agricultural Science Shanghai 200241 China

**Keywords:** baicalin, broiler, immune homeostasis, gut barrier, gut microbiota

## Abstract

The deoxynivalenol (DON)-contaminated feeds can impair chicken gut barrier function, disturb the balance of the intestinal microbiota, decrease chicken growth performance and cause major economic loss. With the aim of investigating the ameliorating effects of baicalin on broiler intestinal barrier damage and gut microbiota dysbiosis induced by DON, a total of 150 Arbor Acres broilers are used in the present study. The morphological damage to the duodenum, jejunum, and ileum caused by DON is reversed by treatment with different doses of baicalin, and the expression of tight junction proteins (ZO-1, claudin-1, and occludin) is also significantly increased in the baicalin-treated groups. Moreover, the disturbance of the intestinal microbiota caused by DON-contaminated feed is altered by baicalin treatment. In particular, compared with those in the DON group, the relative abundances of *Lactobacillus*, *Lachnoclostridium*, *Ruminiclostridium* and other beneficial microbes in the baicalin-treated groups are significantly greater. However, the percentage of *unclassified_f__Lachnospiraceae* in the baicalin-treated groups is significantly decreased in the DON group. Overall, the current results demonstrate that different doses of baicalin can improve broiler intestinal barrier function and the ameliorating effects on broiler intestinal barrier damage may be related to modulations of the intestinal microbiota.

## Introduction

Traditional Chinese medicinal herbs have been applied to treat multiple kinds of diseases for several thousand years. In particular, the significant curing effect of Chinese medicinal herbs might be related to the regulation on the immune homeostasis [1]. *Scutellaria baicalensis* Georgi is a commonly used traditional medicinal plant, and the abundant components of baicalin and other polyphenolic compounds have been proven to be able to treat digestive system diseases effectively [2]. The functional ingredients of *S*. *baicalensis* Georgi could be applied to treat ulcerative colitis through its ability to repair intestinal barrier function [3]. Importantly, baicalin can protect the gastrointestinal tract barrier through regulating tight junction permeation and activating regulatory T-cell differentiation [4]. Previous studies have also shown that baicalin treatment can modulate the composition of the intestinal microbiome and decrease the expression of proinflammatory cytokines (such as interleukin-1, interleukin-6, and tumor necrosis factor) [ 5, 6]. Therefore, baicalin, which was isolated from *S*. *baicalensis* Georgi root, could be explored as a promising candidate for repairing gastrointestinal tract damage through modulation of the gut microbiota.

Feeds containing deoxynivalenol (DON) might cause serious health threats to livestock and humans when DON is consumed in a certain amount. *Fusarium* species can induce *Fusarium* head blight (FHB) in wheat and other crops, and mycotoxins produced by *Fusarium* species can contaminate grains, which are used as feed ingredients [7]. Mycotoxins produced by cereal crops include DON, zearalenone (ZEN), ochratoxin A (OTA), fumonisin B1 (FB1), aflatoxin B1 (AFB1), and patulin (PAT). In particular, DON-contaminated feeds can impair chicken gut barrier permeability and disturb intestinal immunity, ultimately leading to feed refusal and decreased growth performance [8]. The acute toxicological effects of DON are related to increased inflammatory markers, activated regulatory T cells and dendritic cells in mesenteric lymph nodes, and histomorphological impairments from the duodenum to the colon [9]. Moreover, chronic oral administration of DON-contaminated feeds could induce gut microbial dysbiosis and cause intestinal disorders [10]. Payros *et al* . [11] demonstrated that the intestinal DNA damage induced by colibactin-producing *Escherichia coli* could be exacerbated by DON, which might be related to the disrupted balance of the intestinal microbiota.

There are more than 60 billion chickens in the world, and they can provide a large amount of meat and eggs for people in many countries [ 12, 13]. The gut microbiome that inhabits the chicken intestinal tract plays a critical role in providing nutritional supplies, maintaining organ development, increasing fat deposition, and regulating immune homeostasis. A comparison of the microbial communities of the gut microbiota in embryos, chicks, and maternal hens revealed that the establishment and inheritance of avian embryo gut microbes are associated with maternal hens [14]. Previous studies have shown that the avian microbiome in the small intestine, caeca, large intestine and cloaca varies during different growing periods [ 15, 16]. In fact, chicken gut microbial communities can be influenced by host genetics, feed components, the living environment and other factors [ 17‒ 19]. By producing lactic acid, short-chain fatty acids, and other antibacterial metabolites, the gut microbiota can protect the chicken mucosal immune system and inhibit the colonization of invading opportunistic pathogens [20]. Therefore, the chicken gut microbiota could be applied as a novel treatment target to protect the gut barrier and regulate immune function [ 21 , 22].

In the present study, different doses of baicalin were tested for their ability to treat broiler chickens’ gut barrier damage caused by DON-contaminated feed. The growth performance, gut barrier function and gut microbiota of the broiler chickens were investigated and analyzed.

## Materials and Methods

### Experimental design, animals and feeds

A total of 150 healthy Arbor Acres (AA) broilers were purchased from a commercial hatchery and were randomly divided into 5 different groups (the control group, the DON group, and the high, medium and low dose baicalin groups), and each group had 30 chicks. The detailed method of preparing the DON-contaminated feed was described in our previous study [23]. Baicalin was purchased from Nanjing Jiancheng Bioengineering Institute (Nanjing, China). The 5 feeding groups were treated as follows: basal diet, basal diet supplemented with DON (10 mg/kg), basal diet supplemented with DON (10 mg/kg) and a high dose of baicalin (450 mg/kg), basal diet supplemented with DON (10 mg/kg) and medium dose of baicalin (300 mg/kg), basal diet supplemented with DON (10 mg/kg) and low dose of baicalin (150 mg/kg). The ingredients and composition of the basal diet are shown in Table 1.

**Table:** **Table 1** Ingredients and nutrient composition of the basal diets

Ingredients	Percentage (%)	Nutrient composition	Percentage (%)
Corn	61.17	Metabolism energy, (MJ/kg)	12.97
Soybean meal	29.50	Crude protein (%)	20.8
Fishmeal	6.50	Available P (%)	0.45
DL-Met	0.19	Ca (%)	1.02
L-Lys·HCl	0.05	Lys (%)	1.20
Bone meal	1.22	Met+Cys (%)	0.86
Sodium chloride	0.37		
Microelement and vitamin compound premix	1.00		

All the chicks had free access to feed and water during the 14 days. The temperature was maintained at 35°C for the first week and gradually decreased to 27°C in the second week [ 4, 10 ].

### Sampling

At the end of the feeding trial, all the chicks were sacrificed by exsanguination under anesthesia. Subsequently, samples from the duodenum, jejunum, and ileum were collected [21]. All the animal experiments were approved by the Animal Care and Use Committee of Shanxi Agricultural University (2017081).

### The measurement of growth performances

All the chicks were weighed on days 1 and 14. Growth performance was measured by the calculated average daily gain (ADG) and average daily feed intake (ADFI), and the feed conversion ratio (FCR) was calculated as the ratio of the ADFI to the ADG (F/G) [ 3, 24].

### Histological observation

Hematoxylin and eosin (H&E) staining was performed according to methods described in previous studies [ 21, 23]. In detail, tissue samples from the duodenum, jejunum, and ileum (1 cm ^2^) were collected and fixed in 10% neutral-buffered formalin, processed, trimmed, and embedded in paraffin. The morphological changes were observed under a light microscope and scored using ImageJ software (National Institutes of Health, Bethesda, USA).

### Evaluation of intestinal barrier function

The tissue mRNA was detected by real-time RT-PCR using a Rotor-Gene Q2 plex system (Qiagen, Hilden, Germany), and the sequences of primers used were described in a previous study [23]. The protein expression levels of occludin and claudin-1 were measured via western blot analysis. Briefly, the extracted proteins were quantified by a BCA assay and separated via sodium dodecyl sulphate‒polyacrylamide gel electrophoresis (SDS-PAGE). The isolated proteins were subsequently transferred onto a polyvinylidene fluoride (PVDF) membrane (Millipore, Bedford, USA) and blocked with 5% skim milk for 2 h at room temperature. After incubation with primary antibodies against occludin (1:2000), claudin-1 (1:2000), or β-actin (1:5000) (Cell Signaling Technology, Danvers, USA) at 4°C overnight, the membranes were incubated with horseradish peroxidase (HRP)-conjugated goat anti-mouse and goat anti-rabbit IgG (Cell Signaling Technology) for 2 h at room temperature. The membranes were subsequently treated with enhanced chemiluminescence (ECL) reagent (CWbio Inc., Taizhou, China) and then exposed to X-ray film. The images of protein bands were captured and analyzed with Image-Pro Plus software [ 3, 4] .

### Gut microbiota profiling with 16S rDNA sequencing

The genomic DNA of the gut microbiota was extracted from the cecal samples using an E.Z.N.A.® soil DNA Kit (Omega Biotek, Norcross, USA) according to the manufacturer’s instructions. The hypervariable region V3-V4 of the bacterial 16S rRNA gene was amplified with the primer pairs 338F (5′-ACTCCTACGGGAGGCAGCAG-3′) and 806R (5′-GGACTACHVGGGTWTCTAAT-3′). PCR amplification of the 16S rRNA gene was performed as follows: initial denaturation at 95°C for 3 min; 27 cycles of denaturation at 95°C for 30 s, annealing at 55°C for 30 s and extension at 72°C for 45 s; and a single extension at 72°C for 10 min, ending at 4°C [25]. The purified amplicons were pooled in equimolar amounts and paired-end sequenced on an Illumina MiSeq PE300 platform (Illumina, San Diego, USA), which was performed by Majorbio Bio-Pharm Technology Co., Ltd. (Shanghai, China). The raw 16S rRNA gene sequencing reads were demultiplexed and quality-filtered by fastp (version 0.20.0). The operational taxonomic units (OTUs) with a 97% similarity cut-off were clustered using UPARSE version 7.1, and the chimeric sequences were identified and removed [ 26, 27 ].

### Statistical analysis

All the results are presented as the mean±SD of triplicate measurements. Differences were analyzed using the Kruskal-Wallis test or one-way ANOVA, and *P*<0.05 was considered statistically significant.

## Results

### The broiler growth performance was improved by baicalin treatment

On day 14, all the chicks from the five groups were weighed and sacrificed, and the broiler’s growth performances were measured. As shown in Table 2, the ADG of the DON group was significantly lower than that of the control group ( *P*<0.05). However, the ADGs of the broilers in the baicalin-treated groups were greater than those in the DON group ( *P*<0.05). The enhancing effects of growth performances in the high and medium dose groups were higher than those in the low dose group. Moreover, the ADFI of the broilers in the DON group was significantly lower than that of the broilers in the control group ( *P*<0.05). Moreover, the ADFIs of the broilers in the baicalin-treated groups were significantly greater than those in the DON group ( *P*<0.05), and the ADFIs of the broilers in the high and medium dose groups were much greater than those in the low dose group. The calculated F/G of the DON group was lower than that of the control group but was greater after baicalin treatment. Overall, the growth performance of the broilers in the DON group was significantly decreased but was significantly improved by treatment with different doses of baicalin.

**Table:** **Table 2** The growth performances of broilers

Item	ADG (g)	ADFI (g)	F/G
Control	43.17±3.98	56.17±3.05	1.32±0.17
DON	30.08±2.56*	35.50±2.75*	1.19±0.16*
High dose	43.92±3.99 ^#^	56.42±3.59 ^#^	1.30±0.15
Medium dose	43.08±3.62 ^#^	56.08±3.28 ^#^	1.31±0.14
Low dose	40.17±3.00 ^#^	54.00±2.86 ^#^	1.35±0.11

Data are shown as the mean±SD. * *P*<0.05 vs the Control group, ^#^ *P*<0.05 vs the DON group.

### The morphological damages in broiler chicken gut was repaired by baicalin

**Figure 1:**
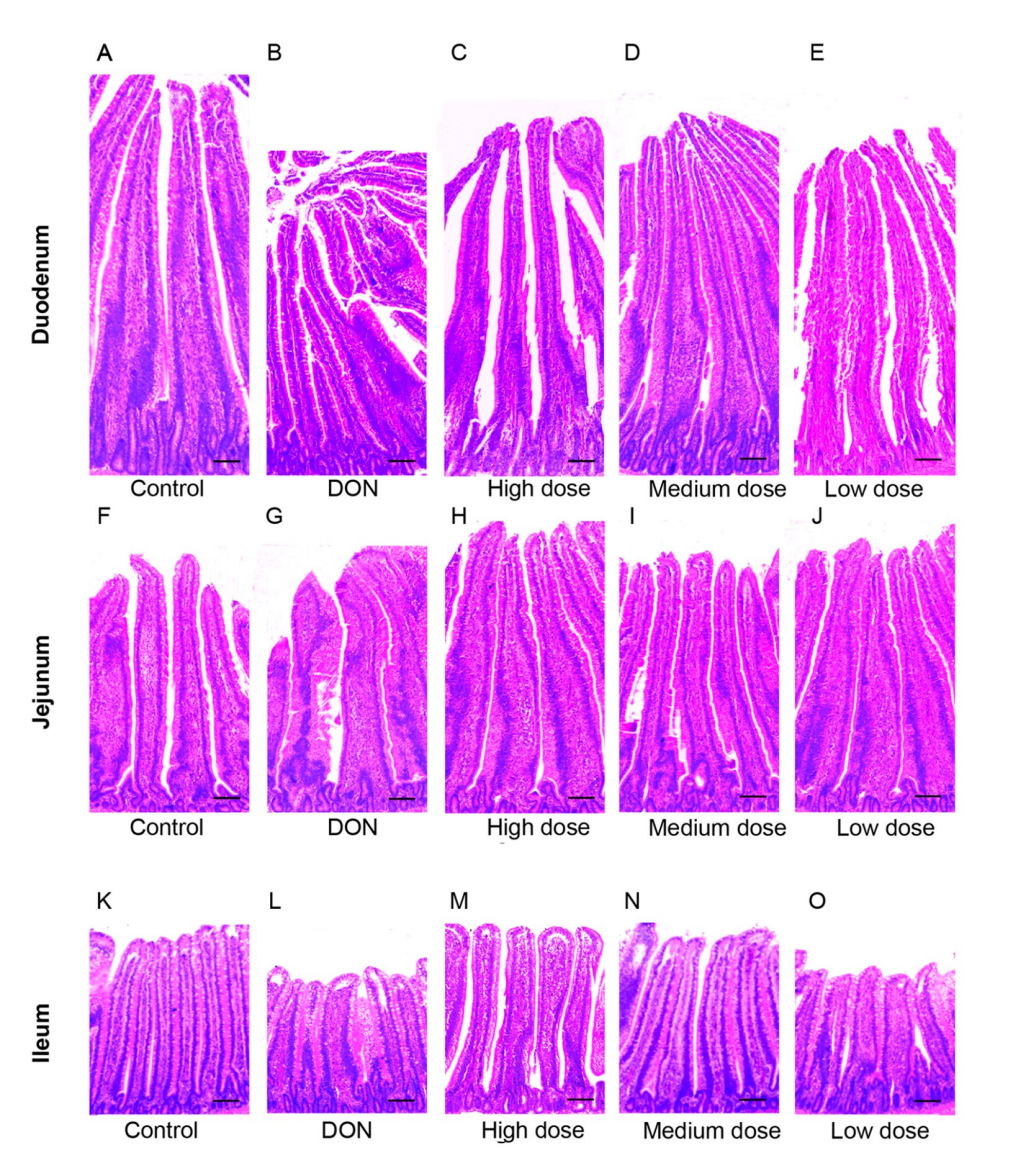
Figure 1 The protective effect of baicalin on the morphological damage to the broiler small intestine caused by DON (A‒E) Morphological changes in the broiler duodenum sections. (F‒J) Morphological changes in the jejunum sections of the broilers. (K‒O) Morphological changes in the ileum sections of the broilers. Scale bar: 100 μm.

Histological changes in the duodenum, jejunum, and ileum of the broilers were examined via H&E staining. As shown in Figure 1, obvious pathological damage caused by the DON-contaminated diet was observed. However, the morphological damage in the duodenum, jejunum, and ileum sections was reversed in the low, medium, and high dose baicalin treatment groups.

### The broiler intestinal barrier function was protected by baicalin treatment

**Figure 2:**
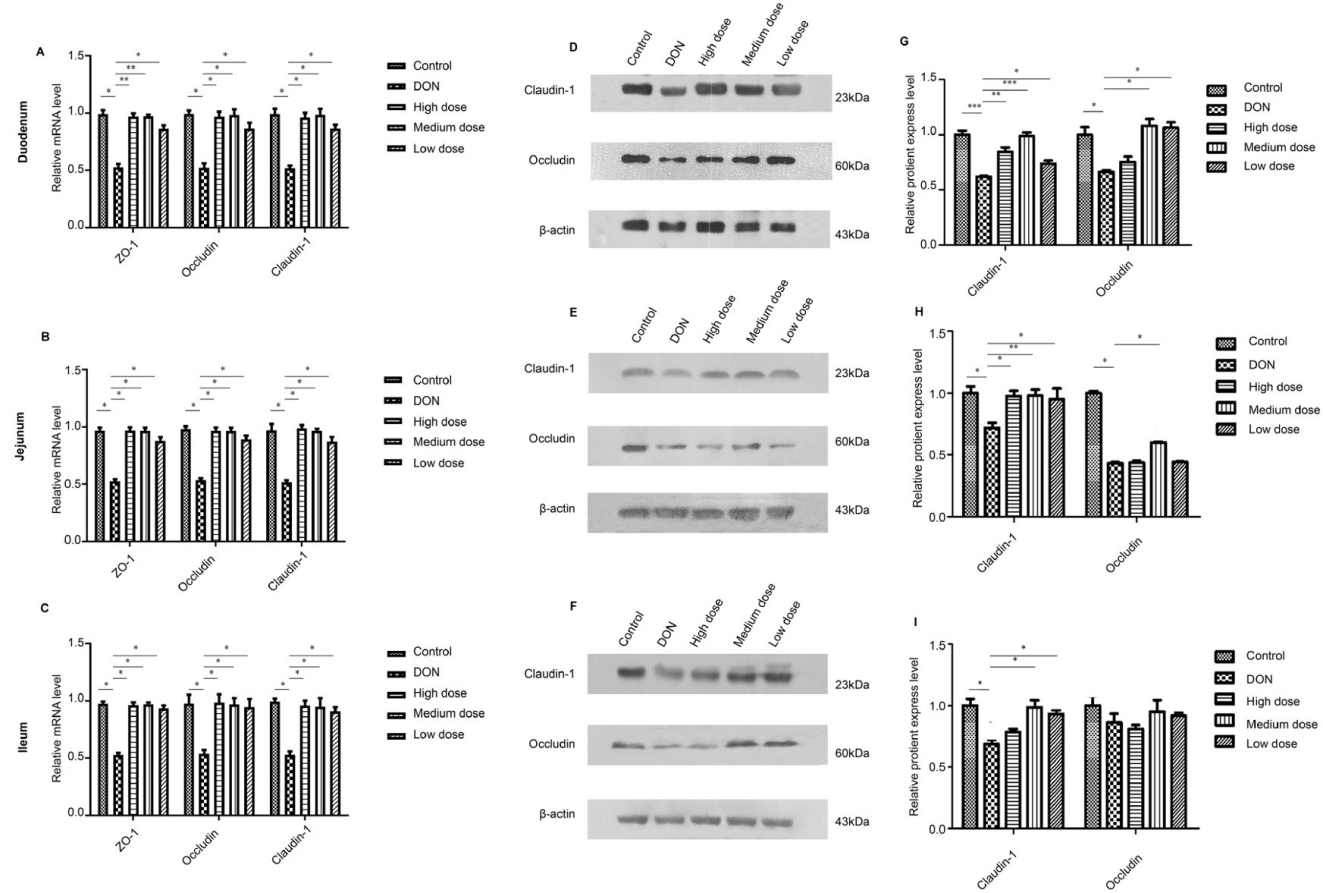
Figure 2 The protective effects of baicalin on broiler gut barrier functions (A‒C) The results of qRT-PCR. (D‒I) The results of western blot analysis showing the protein expression levels of claudin-1 (D) and occludin (G) in broiler duodenum sections; the protein expression levels of claudin-1 (E) and occludin (H) in broiler jejunum sections; and the protein expression levels of claudin-1 (F) and occludin (I) in ileum sections. *P<0.05, **P<0.01, ***P<0.001.

The intestinal barrier functions of the broilers were measured by examining the expressions of tight junction proteins (ZO-1, claudin-1, and occludin). Compared to those in the control group, the mRNA expressions of the ZO-1, claudin-1, and occludin were significantly decreased in the DON group ( Figure 2A‒C). However, the mRNA expressions of the ZO-1, claudin-1, and occludin were significantly increased in the low, medium, and high dose baicalin-treated groups when compared with those in the DON group ( *P*<0.05).

As shown in Figure 2D‒I, the protein expressions of claudin-1 and occludin in the DON group were downregulated when compared to those in the control group. However, treatment with the low, medium, and high doses of baicalin could obviously increase the expressions of claudin-1 and occludin proteins ( *P*<0.05). Our results proved that baicalin treatment could obviously ameliorate the DON-induced destructions of broiler’s intestinal permeability and protect intestinal barrier functions.

### Microbial diversity of the broiler gut microbiota was altered by baicalin treatment

**Figure 3:**
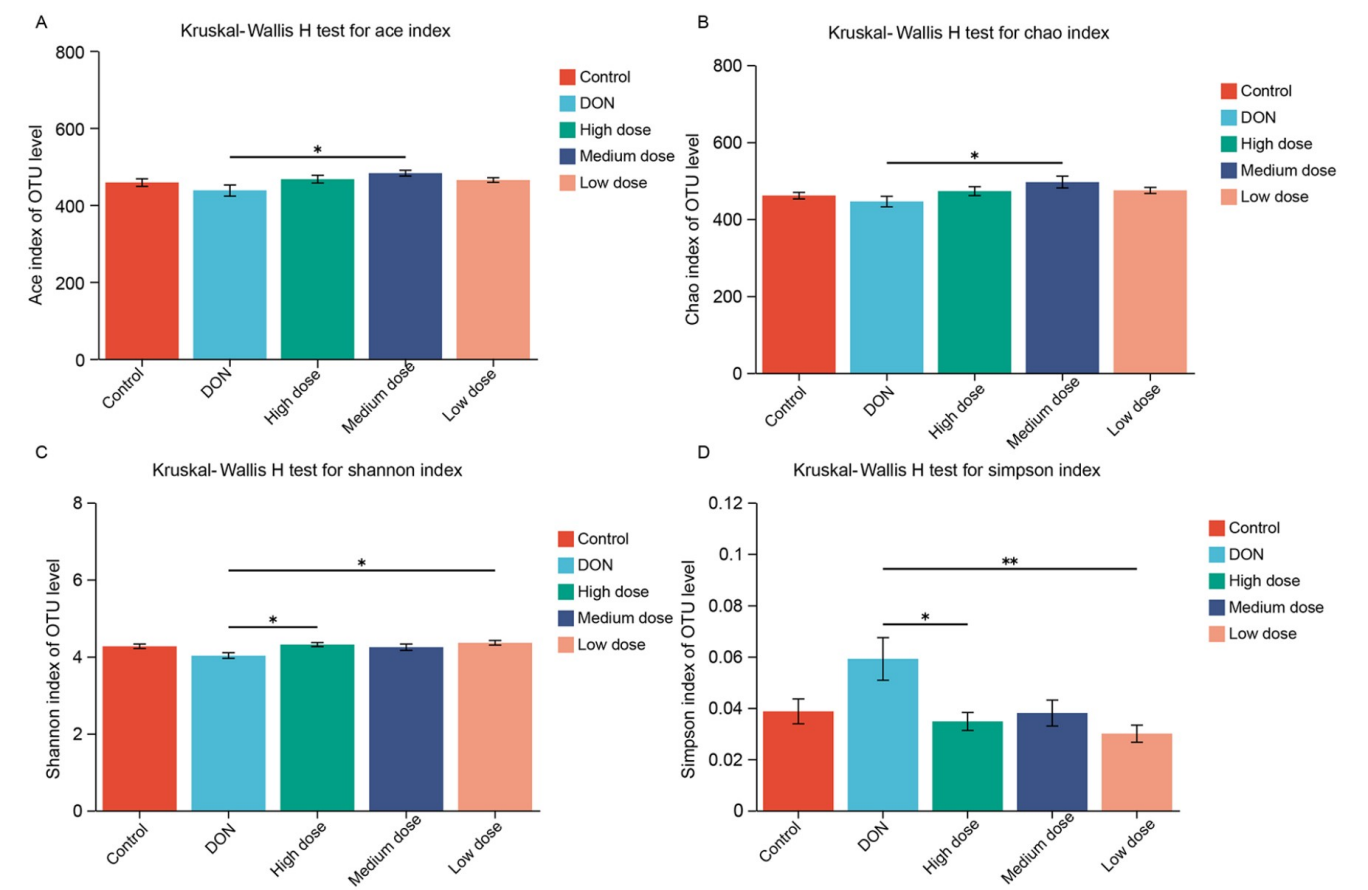
Figure 3 Alpha diversity analysis of the broiler intestinal microbiota (A,B) The richness estimators ACE (A) and Chao (B). The diversity indices Shannon (C) and Simpson (D). *P<0.05, **P<0.01.

All the sequenced raw data were filtered into high-quality reads, and a total of 1,778,698 quality-filtered sequences were obtained, with an average length of 408 bp ( Table 3). The alpha diversities of the broiler gut microbiome were analyzed using the Ace, Chao, Shannon, and Simpson indices, respectively ( Figure 3).

**Table:** **Table 3** Summary of sequenced data of the broilers’ intestinal microbiota

Amplified region	Samples	Sequences	Bases (bp)	Average length
338F_806R	30	1,778,698	725,061,066	408

The Ace and Chao indices indicated that the abundance of the broiler’s gut microbiome in the DON group was lower than that in the control group, but the abundances of the broiler’s gut microbiome in the baicalin-treated groups were much higher than that of the DON group, especially in the medium-dose baicalin group ( *P*<0.05). The Shannon and Simpson indices indicated that the diversity of broiler’s gut microbiome in the DON group was also lower than that in the control group. However, the diversities of broiler’s gut microbiome in the low, medium, and high dose baicalin treatment groups was significantly greater than that in the DON group. Therefore, the richness and diversity of the broiler’s gut microbiome in the DON group were decreased when compared to that in the control group but were increased by the baicalin treatment.

**Figure 4:**
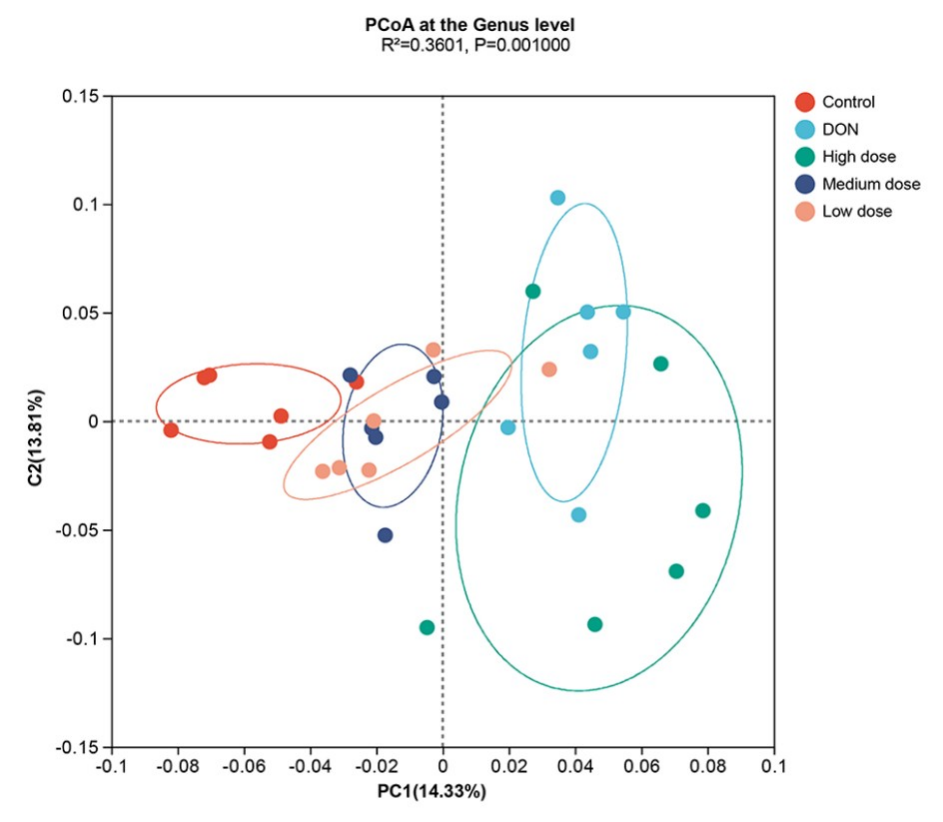
Figure 4 PcoA of the cecal flora in broilers The different experiment groups were segregated into different clusters.

The beta diversity was demonstrated by principal coordinate analysis (PCoA), and the weighted UniFrac distance revealed that samples in the five different groups were segregated into different clusters ( Figure 4).

### The microbial profiles of the broiler intestinal microbiota

**Figure 5:**
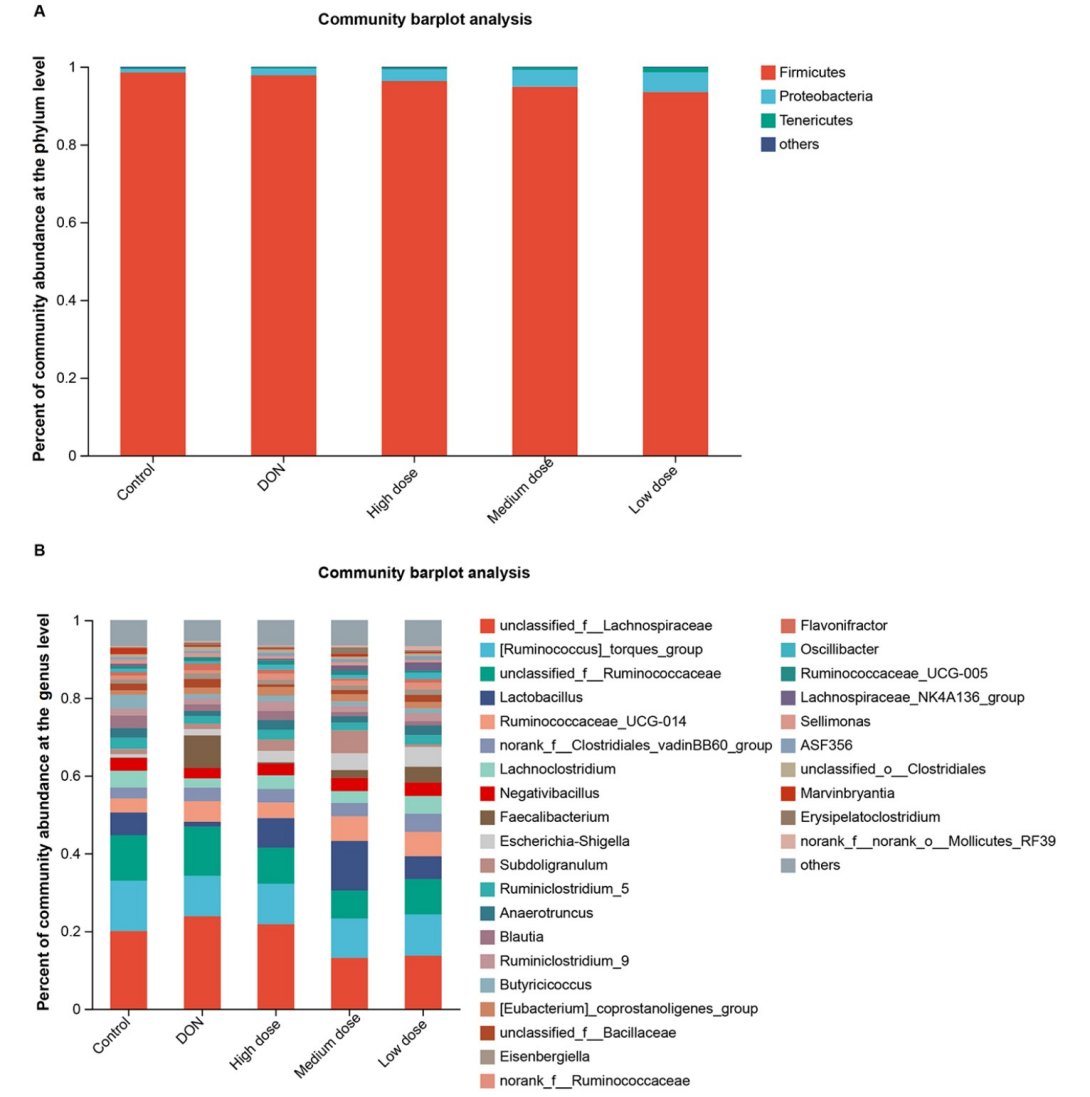
Figure 5 The bacterial communities of the broiler cecal flora at the phylum and genus levels (A) Phylum level. (B) Genus level. Fewer than 1% of the taxa were merged into others.

The RDP classifier was used to assign the bacterial taxonomic compositions at the phylum level and the genus level ( Figure 5). The most predominant microbes at the phylum level were composed of *Firmicutes* (96.20%), *Proteobacteria* (3.01%), *Tenericutes* (0.58%), and others (0.21%) ( Table 4).

**Table:** **Table 4** The predominant taxa at the phylum level

Phyla	Control	DON	Low dose	Medium dose	High dose	Average
*Firmicutes*	98.53%	97.80%	93.49%	94.86%	96.33%	96.17%
*Proteobacteria*	0.88%	1.67%	5.11%	4.37%	3.01%	3.01%
*Tenericutes*	0.31%	0.42%	1.19%	0.61%	0.38%	0.63%
Others	0.28%	0.11%	0.21%	0.16%	0.28%	0.19%

**Figure 6:**
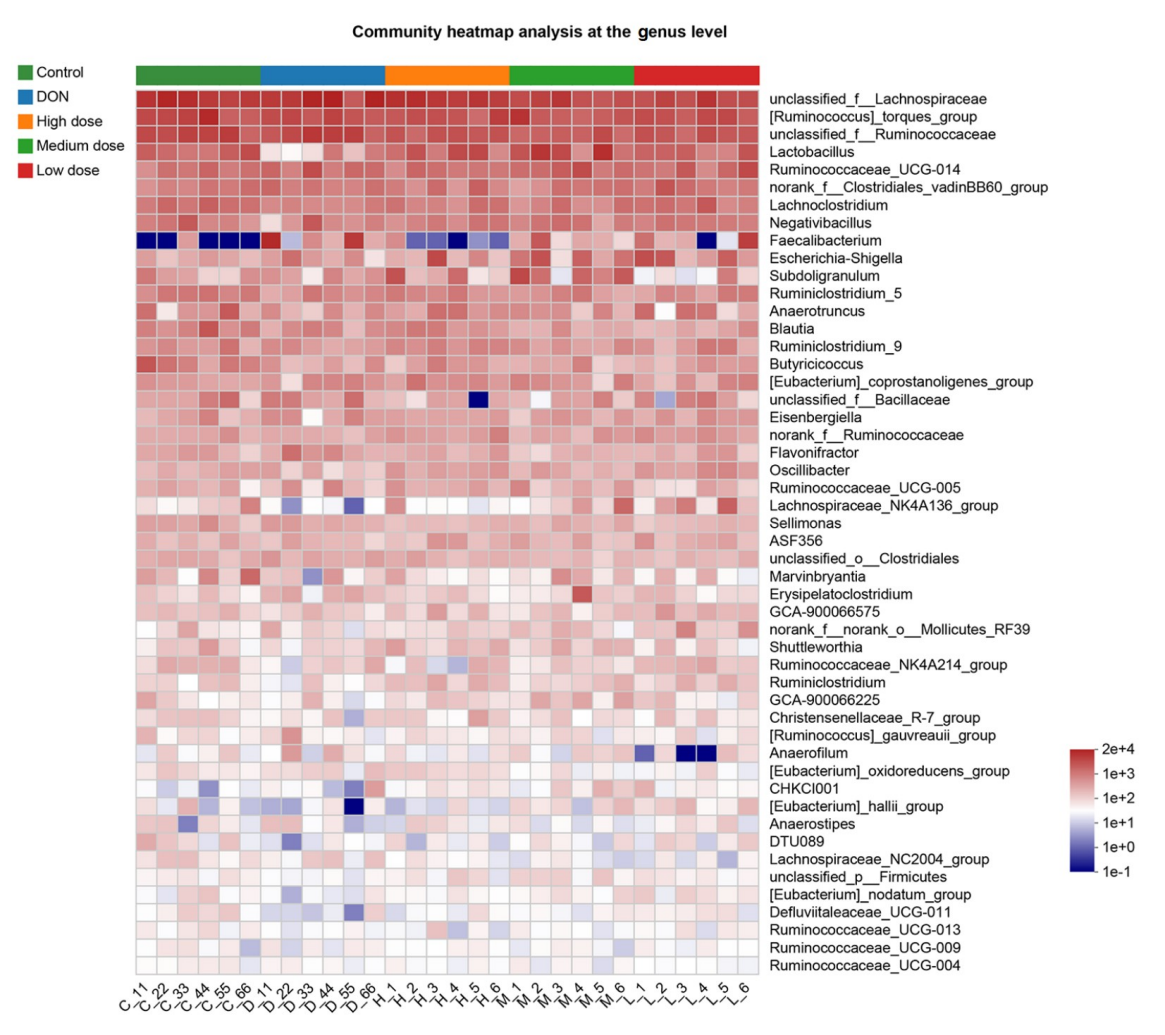
Figure 6 Heatmap analysis of the microbial communities at the genus level The colors of the spots correspond to the normalized and log-transformed relative abundances of the OTUs. The genus names of the OTUs are shown on the right.

The most predominant microbes at the genus level were composed of *unclassified_f__Lachnospiraceae* (18.48%), *[Ruminococcus]_torques_group* (10.92%), *unclassified_f__Ruminococcaceae* (9.95%), *Lactobacillus* (6.69%), *Ruminococcaceae_UCG-014* (5.08%), *norank_f__ Clostridiales_vadinBB60_group* (3.61%), *Lachnoclostridium* (3.53%), *Negativibacillus* (3.17%), *Faecalibacterium* (3.00%), *Escherichia-Shigella* (2.98%), *Subdoligranulum* (2.42%), *Ruminiclostridium_5* (2.35%), *Anaerotruncus* (2.11%), *Blautia* (1.90%), Ruminiclostridium_9 (1.84%), *[Eubacterium] _coprostanoligenes_group* (1.73%), *Butyricicoccus* (1.70%), *unclassified_f__Bacillaceae* (1.52%), *Eisenbergiella* (1.26%), *norank_f__Ruminococcaceae* (1.24%), *Flavonifractor* (1.05%), *Oscillibacter* (1.05%), *Lachnospiraceae_NK4A136_group* (0.90%), *Ruminococcaceae_UCG-005* (0.88%), *Sellimonas* (0.82%), *ASF356* (0.81%), *unclassified_o__Clostridiales* (0.79%), *Erysipelatoclostridium* (0.68%), *Marvinbryantia* (0.68%), *norank_f__norank_o__Mollicutes_RF39* (0.50%) and others (6.32%) ( Table 5). At the genus level, the microbial communities in the five experimental groups were significantly different ( Figure 5B). A heatmap was generated to display the predominant genera, and the color of the spots in the panel indicates the relative abundance in each sample ( Figure 6).

**Table:** **Table 5** The predominant taxa at the genus level

Genera	Control	DON	Low dose	Medium dose	High dose	Average
*unclassified_f__Lachnospiraceae*	20.03%	23.82%	13.68%	13.12%	21.76%	17.66%
*[Ruminococcus]_torques_group*	12.96%	10.39%	10.67%	10.11%	10.45%	11.03%
*unclassified_f__Ruminococcaceae*	11.68%	12.65%	9.03%	7.21%	9.21%	10.14%
*Lactobacillus*	5.85%	1.28%	5.92%	12.76%	7.65%	6.45%
*Ruminococcaceae_UCG-014*	3.57%	5.30%	6.23%	6.34%	3.97%	5.36%
*norank_f__Clostridiales_vadinBB60_group*	2.89%	3.51%	4.66%	3.42%	3.55%	3.62%
*Lachnoclostridium*	4.27%	2.31%	4.56%	3.06%	3.45%	3.55%
*Negativibacillus*	3.24%	2.69%	3.50%	3.38%	3.05%	3.20%
*Faecalibacterium*	0.20%	8.40%	4.04%	2.01%	0.35%	3.66%
*Escherichia-Shigella*	0.85%	1.63%	5.09%	4.35%	2.98%	2.98%
*Subdoligranulum*	1.28%	1.35%	0.69%	5.89%	2.87%	2.30%
*Ruminiclostridium_5*	2.92%	1.98%	2.33%	2.00%	2.51%	2.31%
*Anaerotruncus*	2.52%	1.33%	2.56%	1.64%	2.52%	2.01%
*Blautia*	3.23%	1.74%	1.10%	1.07%	2.34%	1.78%
*Ruminiclostridium_9*	1.75%	1.44%	1.99%	1.61%	2.43%	1.70%
*[Eubacterium]_coprostanoligenes_group*	1.15%	1.76%	1.64%	1.91%	2.20%	1.62%
*Butyricicoccus*	3.55%	1.11%	1.23%	1.10%	1.51%	1.75%
*unclassified_f__Bacillaceae*	1.77%	2.20%	1.86%	1.08%	0.68%	1.73%
*Eisenbergiella*	1.04%	1.42%	1.47%	1.18%	1.17%	1.28%
*norank_f__Ruminococcaceae*	0.98%	0.83%	1.62%	1.15%	1.63%	1.14%
*Oscillibacter*	0.80%	0.60%	1.67%	0.88%	1.32%	0.99%
*Flavonifractor*	0.90%	1.75%	0.98%	0.63%	0.98%	1.06%
*Lachnospiraceae_NK4A136_group*	0.61%	0.06%	2.08%	1.35%	0.41%	1.03%
*Ruminococcaceae_UCG-005*	0.71%	0.97%	0.54%	1.12%	1.07%	0.84%
*Sellimonas*	1.01%	0.99%	0.62%	0.83%	0.62%	0.86%
*ASF356*	0.66%	0.62%	1.01%	0.80%	0.92%	0.78%
*unclassified_o__Clostridiales*	0.78%	1.00%	0.68%	0.63%	0.85%	0.77%
*Erysipelatoclostridium*	0.31%	0.68%	0.41%	1.67%	0.34%	0.77%
*Marvinbryantia*	1.53%	0.47%	0.36%	0.71%	0.33%	0.77%
*norank_f__norank_o__Mollicutes_RF39*	0.29%	0.33%	1.07%	0.48%	0.31%	0.54%
Others	6.65%	5.39%	6.72%	6.51%	6.54%	6.32%

**Figure 7:**
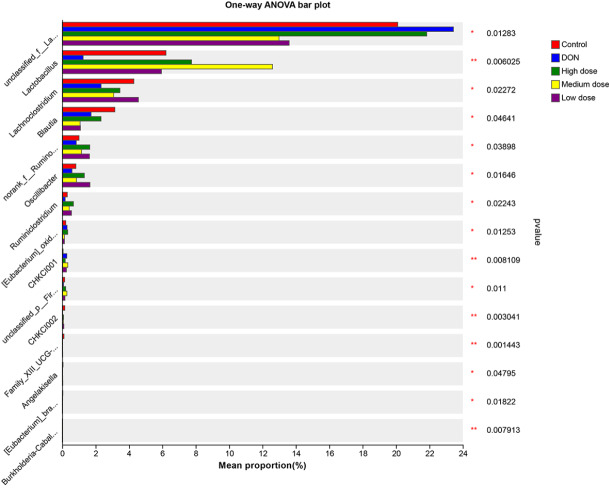
Figure 7 Comparisons of the different microbial communities at the genus level The ordinate indicates the bacterial name, and the abscissa indicates the percentage value. *P<0.05, **P<0.01.

To compare the relative abundances of the predominant genera among the five groups, one-way ANOVA was used to analyze the differences in the mean percentages ( Figure 7). The percentages of *Lactobacillus*, *Lachnoclostridium*, and *Ruminiclostridium* in the DON group were significantly lower than those in the control group but were significantly greater in the baicalin-treated groups ( *P*<0.05). However, compared with that in the control group, the percentage of *unclassified_f__Lachnospiraceae* in the DON group was significantly greater but was significantly lower in the baicalin-treated groups. Therefore, the genera that were significantly different among the five groups might play critical roles in the reparative effects of baicalin treatment.

### PICRUSt functional prediction

**Figure 8:**
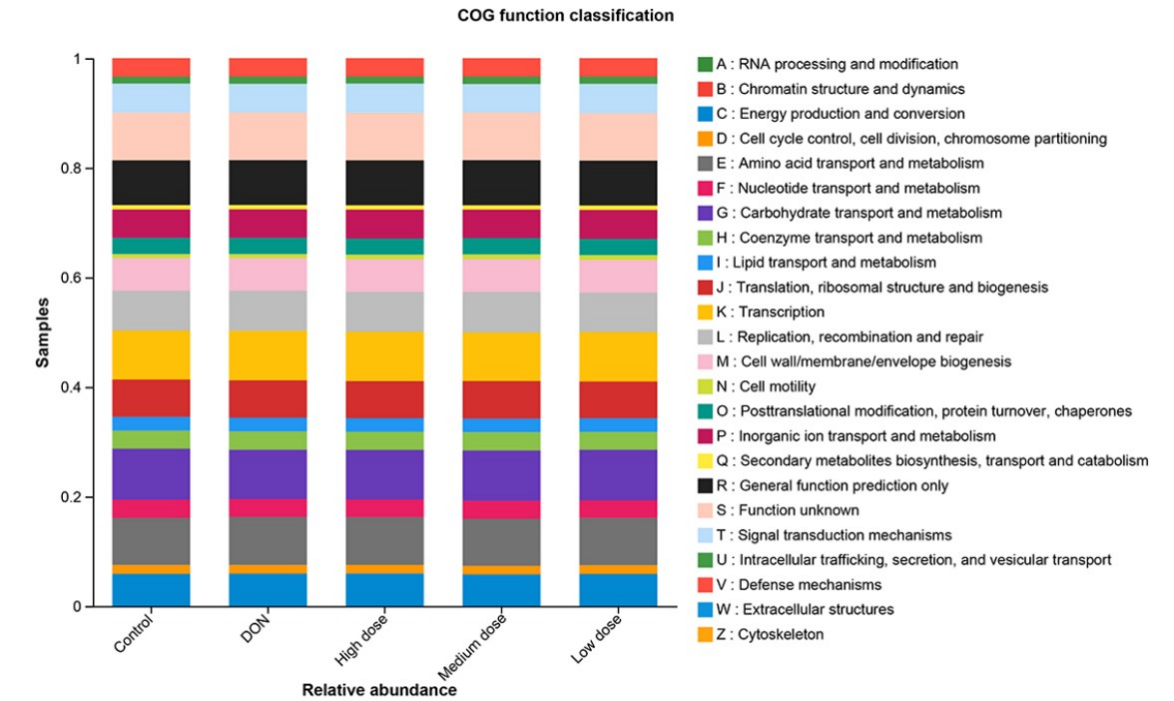
Figure 8 The predicted PICRUSt functions The signaling pathways related to gastrointestinal function and metabolism were calculated.

The predicted functions were calculated based on PICRUSt in the EggNOG database (evolutionary genealogy of genes: Non-supervised Orthologous Groups, http://eggnog.embl.de/), and 23 pathways associated with broiler intestinal diseases were identified ( Figure 8). The microbial genes involved in RNA processing and modification, extracellular structures, nucleotide transport and metabolism, energy production and conversion, cell motility, carbohydrate transport and metabolism, lipid transport and metabolism, coenzyme transport and metabolism, defense mechanisms, amino acid transport and metabolism were more common in the baicalin-treated groups than in the DON group. These predicted signaling pathways might be related to gastrointestinal function and metabolism in broilers.

## Discussion

As a plant-derived antibacterial compound, baicalin is well known for its protective effect against gut inflammatory injury, and the physiological mechanism might be related to the modulation of the gut microbiota [28]. Importantly, the immunoregulatory function of plant flavonoids is associated mainly with bioactive compounds that are conserved by the gut microbiome [29]. In addition, previous studies have proven that baicalin can modulate the gut microbiome composition and regulate the intestinal metabolic function of animals with diarrhea [ 27, 30]. In this study, the growth performance, gut barrier function and gut microbiota of broiler chickens were investigated.

The administration of DON-contaminated diets could induce intestinal epithelial cell cytotoxicity and apoptosis, and subsequently cause gastrointestinal inflammation and destruction of the gut barrier integrity in broiler chickens [ 9‒ 11, 31]. The broiler ADG in the DON group was significantly lower than that in the control group. However, compared with those in the DON group, the ADGs in the baicalin-treated groups were significantly greater ( Table 2). Moreover, compared with those in the DON group, the ADFIs in the baicalin-treated group were also significantly greater ( *P*<0.05). Consistent with the findings of previous studies, the present study revealed that DON-contaminated feeds could disturb broiler chickens’ intestinal digestive function and decrease growth performance [ 8, 23]. In addition, the calculated F/G values in the baicalin-treated groups were greater than those in the DON group. Therefore, these data indicated that baicalin treatment could improve DON-induced feed refusal and improve broiler chicken growth.

As shown in Figure 1, obvious pathological damage caused by DON was observed. However, the morphological damage to the duodenum, jejunum, and ileum was obviously alleviated in the low, medium, and high-dose baicalin treatment groups. The protective effects of baicalin treatments on DON-induced destruction of gut permeability and intestinal barrier injuries were related to the expressions of tight junction proteins (ZO-1, claudin-1, and occludin). The mRNA expression levels of ZO-1, claudin-1, and occludin were significantly greater in the low, medium, and high dose baicalin-treated groups than in the DON group ( Figure 2A‒C). Moreover, treatment with baicalin at low, medium, or high doses markedly upregulated the protein expressions of claudin-1 and occludin ( Figure 2D‒I). The intestinal barrier damage induced by DON is likely related to the excessive production of reactive oxygen species (ROS) and the activation of critical kinases involved in cellular proliferation, differentiation, and apoptosis [ 31, 32]. Previous studies have also shown that altered gut barrier function is related to intestinal oxidative stress and inflammatory responses [ 33, 34]. Moreover, the free radical scavenging activity of baicalin could attenuate inflammation by acting on neutrophils and regulating the production of inflammatory cytokines [35]. Therefore, the present study demonstrated that baicalin treatment could ameliorate the DON-induced destruction of intestinal barrier permeability through enhancing the expression of tight junction proteins (claudin-1, occludin, and ZO-1).

The commensal gut microbiota, which inhabits the broiler intestinal tract, plays an important role in maintaining immune homeostasis. Our results demonstrated that the alpha diversity of the broiler gut microbiome was decreased by the DON-contaminated diet, but baicalin treatment restored the richness and diversity of the broiler gut microbiota ( Figure 3). Moreover, the beta diversity analysis revealed that the five experimental groups were obviously separated into different community clusters ( Figure 4). Therefore, the gut microbial diversity, which was disrupted by the DON-contaminated diet, could be restored through baicalin treatment. Moreover, the bacterial taxonomic analyses at the phylum and genus levels revealed that the microbial compositions in the five experimental groups were quite different ( Figures 5 and 6). The most predominant microbes at the phylum and genus levels are shown in Tables 4 and 5, respectively. One-way ANOVA was used to compare the taxonomic differences among the five groups at the genus level ( Figure 7). Compared to those in the control group, the relative abundances of *Lactobacillus*, *Lachnoclostridium*, and *Ruminiclostridium* in the DON group were significantly lower but were significantly greater in the baicalin-treated groups. The present study indicated that baicalin treatment could obviously increase the abundance of *Lactobacillus*, *Lachnoclostridium*, and *Ruminiclostridium* in the broiler gut. In fact, *Lactobacillus* species are commonly considered to be beneficial microbes because most members of the *Lactobacillus* genus can stimulate the immune system, modulate the gut microbiota, and prevent oxidative damage [ 36‒ 38]. Moreover, the bacterial members *Lactobacillus*, *Lachnoclostridium*, and *Ruminiclostridium* can produce various kinds of beneficial metabolites (such as lactic acid, short-chain fatty acids, and bacteriocins), and these antibacterial metabolites can inhibit invading opportunistic pathogens. However, the relative abundance of *unclassified_f__Lachnospiraceae* in the baicalin *-*treated groups was significantly lower than that in the DON group. These genera, which were significantly different among the five groups, might play critical roles in the reparative effects of baicalin treatment. Commonly, the bioavailability of baicalin and other plant-derived polyphenols is difficult to demonstrate due to its low absorbance [39]. When these herbal medicinal products reach the colon, the gut microbiota can catabolize these ingested phenolic compounds and release additional active metabolites [40]. On the other hand, baicalin could also shape the structure of the gut microbiota and improve intestinal epithelial homeostasis [41]. By enhancing the relative abundances of beneficial microbes and decreasing the relative abundances of harmful bacteria, the altered gut microbiota could protect against intestinal barrier dysfunction and regulate intestinal inflammation caused by DON exposure [42]. Therefore, the complicated interactions between baicalin and the gut microbiota could ameliorate DON-induced intestinal damage through enhancing the expressions of tight junction proteins.

In conclusion, the current research demonstrated that different doses of baicalin could improve broiler intestinal barrier function, and these ameliorating effects might be related to the modulation of the intestinal microbiota.

## References

[1] Lin TL, Lu CC, Lai WF, Wu TS, Lu JJ, Chen YM, Tzeng CM (2021). Role of gut microbiota in identification of novel TCM-derived active metabolites. Protein Cell.

[2] Dai J, Liang K, Zhao S, Jia W, Liu Y, Wu H, Lv J (2018). Chemoproteomics reveals baicalin activates hepatic CPT1 to ameliorate diet-induced obesity and hepatic steatosis. Proc Natl Acad Sci USA.

[3] Cui L, Guan X, Ding W, Luo Y, Wang W, Bu W, Song J (2021). Scutellaria baicalensis Georgi polysaccharide ameliorates DSS-induced ulcerative colitis by improving intestinal barrier function and modulating gut microbiota. Int J Biol Macromolecules.

[4] Bae MJ, Shin HS, See HJ, Jung SY, Kwon DA, Shon DH (2016). Baicalein induces CD4+Foxp3+ T cells and enhances intestinal barrier function in a mouse model of food allergy. Sci Rep.

[5] Gao L, Li J, Zhou Y, Huang X, Qin X, Du G (2018). Effects of baicalein on cortical proinflammatory cytokines and the intestinal microbiome in senescence accelerated mouse prone 8. ACS Chem Neurosci.

[6] Yang Y, Chen G, Yang Q, Ye J, Cai X, Tsering P, Cheng X (2017). Gut microbiota drives the attenuation of dextran sulphate sodium-induced colitis by Huangqin decoction. Oncotarget.

[7] Wang H, Sun S, Ge W, Zhao L, Hou B, Wang K, Lyu Z (2020). Horizontal gene transfer of
*Fhb7* from fungus underlies
*Fusarium* head blight resistance in wheat. Science.

[8] Robert H, Payros D, Pinton P, Théodorou V, Mercier-Bonin M, Oswald IP (2017). Impact of mycotoxins on the intestine: are mucus and microbiota new targets?. J Toxicol Environ Health Part B.

[9] Vignal C, Djouina M, Pichavant M, Caboche S, Waxin C, Beury D, Hot D (2018). Chronic ingestion of deoxynivalenol at human dietary levels impairs intestinal homeostasis and gut microbiota in mice. Arch Toxicol.

[10] Yang X, Liang S, Guo F, Ren Z, Yang X, Long F (2020). Gut microbiota mediates the protective role of
*Lactobacillus plantarum*in ameliorating deoxynivalenol-induced apoptosis and intestinal inflammation of broiler chickens. Poultry Sci.

[11] Payros D, Dobrindt U, Martin P, Secher T, Bracarense APFL, Boury M, Laffitte J (2017). The food contaminant deoxynivalenol exacerbates the genotoxicity of gut microbiota. mBio.

[12] Feng Y, Wang Y, Zhu B, Gao GF, Guo Y, Hu Y (2021). Metagenome-assembled genomes and gene catalog from the chicken gut microbiome aid in deciphering antibiotic resistomes. Commun Biol.

[13] Glendinning L, Stewart RD, Pallen MJ, Watson KA, Watson M (2020). Assembly of hundreds of novel bacterial genomes from the chicken caecum. Genome Biol.

[14] Ding J, Dai R, Yang L, He C, Xu K, Liu S, Zhao W (2017). Inheritance and establishment of gut microbiota in chickens. Front Microbiol.

[15] Wilkinson TJ, Cowan AA, Vallin HE, Onime LA, Oyama LB, Cameron SJ, Gonot C (2017). Characterization of the microbiome along the gastrointestinal tract of growing turkeys. Front Microbiol.

[16] Xiao Y, Xiang Y, Zhou W, Chen J, Li K, Yang H (2017). Microbial community mapping in intestinal tract of broiler chicken. Poultry Sci.

[17] Cui Y, Wang Q, Liu S, Sun R, Zhou Y, Li Y (2017). Age-related variations in intestinal microflora of free-range and caged hens. Front Microbiol.

[18] Wang L, Lilburn M, Yu Z. Intestinal microbiota of broiler chickens as affected by litter management regimens.
*
Front Microbiol
* 2016, 7: 593. https://doi.org/10.3389/fmicb.2016.00593.

[19] McKenna A, Ijaz UZ, Kelly C, Linton M, Sloan WT, Green BD, Lavery U (2020). Impact of industrial production system parameters on chicken microbiomes: mechanisms to improve performance and reduce Campylobacter. Microbiome.

[20] Zenner C, Hitch TCA, Riedel T, Wortmann E, Tiede S, Buhl EM, Abt B (2021). Early-Life immune system maturation in chickens using a synthetic community of cultured gut bacteria. mSystems.

[21] Wang H, Ni X, Qing X, Liu L, Lai J, Khalique A, Li G (2017). Probiotic enhanced intestinal immunity in broilers against subclinical necrotic enteritis. Front Immunol.

[22] Yan W, Sun C, Yuan J, Yang N (2017). Gut metagenomic analysis reveals prominent roles of
*Lactobacillus* and cecal microbiota in chicken feed efficiency. Sci Rep.

[23] Wan S, Sun N, Li H, Khan A, Zheng X, Sun Y, Fan R (2022). Deoxynivalenol damages the intestinal barrier and biota of the broiler chickens. BMC Vet Res.

[24] Qing X, Zeng D, Wang H, Ni X, Liu L, Lai J, Khalique A (2017). Preventing subclinical necrotic enteritis through
*Lactobacillus johnsonii* BS15 by ameliorating lipid metabolism and intestinal microflora in broiler chickens. AMB Expr.

[25] Gao P, Ma C, Sun Z, Wang L, Huang S, Su X, Xu J (2017). Feed-additive probiotics accelerate yet antibiotics delay intestinal microbiota maturation in broiler chicken. Microbiome.

[26] Xu B, Yan Y, Yin B, Zhang L, Qin W, Niu Y, Tang Y (2021). Dietary glycyl-glutamine supplementation ameliorates intestinal integrity, inflammatory response, and oxidative status in association with the gut microbiota in LPS-challenged piglets. Food Funct.

[27] Fu S, Zhuang F, Guo L, Qiu Y, Xiong J, Ye C, Liu Y (2019). Effect of baicalin-aluminum complexes on fecal microbiome in piglets. Int J Mol Sci.

[28] Wang J, Ishfaq M, Li J (2021). Baicalin ameliorates
*Mycoplasma gallisepticum*-induced inflammatory injury in the chicken lung through regulating the intestinal microbiota and phenylalanine metabolism. Food Funct.

[29] Kaakoush NO, Morris MJ (2017). More flavor for flavonoid-based interventions?. Trends Mol Med.

[30] Guo L, He J, Zhang J, Zhang X, Zhang D, Zhou L, Yuan Y (2021). Baicalin-aluminum modulates the broiler gut microbiome. DNA Cell Biol.

[31] Guo F, Wang F, Ma H, Ren Z, Yang X, Yang X (2021). Study on the interactive effect of deoxynivalenol and
*Clostridium perfringens*on the jejunal health of broiler chickens. Poultry Sci.

[32] Peltonen S, Riehokainen J, Pummi K, Peltonen J (2007). Tight junction components occludin, ZO-1, and claudin-1, -4 and -5 in active and healing psoriasis. Br J Dermatol.

[33] Zhai X, Qiu Z, Wang L, Luo Y, He W, Yang J (2022). Possible toxic mechanisms of deoxynivalenol (DON) exposure to intestinal barrier damage and dysbiosis of the gut microbiota in laying hens. Toxins.

[34] Springler A, Hessenberger S, Schatzmayr G, Mayer E (2016). Early activation of MAPK p44/42 is partially involved in DON-induced disruption of the intestinal barrier function and tight junction network. Toxins.

[35] Li L, Cui H, Zhang Y, Xie W, Lin Y, Guo Y, Huang T (2023). Baicalin ameliorates multidrug-resistant
*Pseudomonas aeruginosa* induced pulmonary inflammation in rat via arginine biosynthesis. Biomed Pharmacother.

[36] Wu S, Liu Y, Duan Y, Wang F, Guo F, Yan F, Yang X (2018). Intestinal toxicity of deoxynivalenol is limited by supplementation with
*Lactobacillus plantarum* JM113 and consequentially altered gut microbiota in broiler chickens. J Anim Sci Biotechnol.

[37] Wang L, Liu C, Chen M, Ya T, Huang W, Gao P, Zhang H (2015). A novel
*Lactobacillus plantarum* strain P-8 activates beneficial immune response of broiler chickens. Int Immunopharmacol.

[38] Chen Q, Tong C, Ma S, Zhou L, Zhao L, Zhao X (2017). Involvement of microRNAs in probiotics-induced reduction of the cecal inflammation by
*Salmonella* Typhimurium. Front Immunol.

[39] Espín JC, González-Sarrías A, Tomás-Barberán FA (2017). The gut microbiota: a key factor in the therapeutic effects of (poly) phenols. Biochem Pharmacol.

[40] Li Q, Van de Wiele T (2023). Gut microbiota as a driver of the interindividual variability of cardiometabolic effects from tea polyphenols. Crit Rev Food Sci Nutr.

[41] Wu Z, Huang S, Li T, Li N, Han D, Zhang B, Xu ZZ (2021). Gut microbiota from green tea polyphenol-dosed mice improves intestinal epithelial homeostasis and ameliorates experimental colitis. Microbiome.

[42] Bai Y, Ma K, Li J, Ren Z, Zhang J, Shan A (2022). *Lactobacillus rhamnosus* GG ameliorates DON-induced intestinal damage depending on the enrichment of beneficial bacteria in weaned piglets. J Anim Sci Biotechnol.

